# Patients with type 1 diabetes show signs of vascular dysfunction in response to multiple high-fat meals

**DOI:** 10.1186/1743-7075-11-28

**Published:** 2014-06-13

**Authors:** Mariann I Lassenius, Ville-Petteri Mäkinen, Christopher L Fogarty, Lina Peräneva, Matti Jauhiainen, Pirkko J Pussinen, Marja-Riitta Taskinen, Juha Kirveskari, Outi Vaarala, Janne K Nieminen, Sohvi Hörkkö, Antti J Kangas, Pasi Soininen, Mika Ala-Korpela, Daniel Gordin, Aila J Ahola, Carol Forsblom, Per-Henrik Groop, Markku Lehto

**Affiliations:** 1 Folkhälsan Institute of Genetics, Folkhälsan Research Center, Biomedicum, Helsinki, Finland; 2 Department of Medicine, Division of Nephrology, Helsinki University Central Hospital, Helsinki, Finland; 3 Diabetes & Obesity Research Program, Research Program’s Unit, University of Helsinki, Helsinki, Finland; 4 Department of Integrative Biology and Physiology, University of California, Los Angeles, USA; 5 Public Health Genomics Unit, National Institute for Health and Welfare, Helsinki, Finland; 6 Institute of Dentistry, University of Helsinki, Helsinki, Finland; 7 Heart and Lung Center, Cardiovascular Research Group, HUCH, Helsinki, Finland; 8 Department of Bacteriology, HUSLAB, Helsinki, Finland; 9 Immune Response Unit, Department of Vaccination and Immune Protection, National Institute for Health and Welfare, Helsinki, Finland; 10 Department of Medical Microbiology and Immunology, University of Oulu, and Nordlab, Oulu, Finland; 11 Computational Medicine, Institute of Health Sciences, University of Oulu, Oulu, Finland; 12 NMR Metabolomics Laboratory, School of Pharmacy, University of Eastern Finland, Kuopio, Finland; 13 Oulu University Hospital, Oulu, Finland; 14 Computational Medicine, School of Social and Community Medicine & Medical Research Council Integrative Epidemiology Unit, University of Bristol, Bristol, UK; 15 Baker IDI Heart & Diabetes Institute, Melbourne, Australia

**Keywords:** High-fat diet, Vascular dysfunction, Type 1 diabetes

## Abstract

**Background:**

A high-fat diet promotes postprandial systemic inflammation and metabolic endotoxemia. We investigated the effects of three consecutive high-fat meals on endotoxemia, inflammation, vascular function, and postprandial lipid metabolism in patients with type 1 diabetes.

**Methods:**

Non-diabetic controls (n = 34) and patients with type 1 diabetes (n = 37) were given three high-caloric, fat-containing meals during one day. Blood samples were drawn at fasting (8:00) and every two hours thereafter until 18:00. Applanation tonometry was used to assess changes in the augmentation index during the investigation day.

**Results:**

Three consecutive high-fat meals had only a modest effect on serum LPS-activity levels and inflammatory markers throughout the day in both groups. Of note, patients with type 1 diabetes were unable to decrease the augmentation index in response to the high-fat meals. The most profound effects of the consecutive fat loads were seen in chylomicron and HDL-metabolism. The triglyceride-rich lipoprotein remnant marker, apoB-48, was elevated in patients compared to controls both at fasting (p = 0.014) and postprandially (p = 0.035). The activities of the HDL-associated enzymes PLTP (p < 0.001), and CETP (p = 0.007) were higher and paraoxonase (PON-1) activity, an anti-oxidative enzyme bound to HDL, decreased in patients with type 1 diabetes (p = 0.027).

**Conclusions:**

In response to high-fat meals, early signs of vascular dysfunction alongside accumulation of chylomicron remnants, higher augmentation index, and decreased PON-1 activity were observed in patients with type 1 diabetes. The high-fat meals had no significant impact on postprandial LPS-activity in non-diabetic subjects or patients with type 1 diabetes suggesting that metabolic endotoxemia may be more central in patients with chronic metabolic disturbances such as obesity, type 2 diabetes, or diabetic kidney disease.

## Background

Postprandial lipid accumulation, in the form of triglyceride-rich lipoprotein (TRL) particles, is linked to high-fat meal induced inflammation [1]. Absorption of bacterial endotoxin/lipopolysaccharide (LPS) from the gut is postulated to contribute to the development of chronic inflammation in mammals after a high-fat diet [2,3].

Endotoxins are unique, fat-soluble glycolipids found in the outer membrane of gram-negative bacteria. Systemically, endotoxins are primarily associated with circulating LPS-binding proteins (LBP), the endothelium of blood vessels, and lipoprotein particles. Binding of the LBP/LPS-complex to Toll-like receptor 4 (TLR4) triggers an innate immune response, characterized by cytokine release and immune system activation [4]. Circulating HDL is likely the most important factor involved in systemic endotoxin neutralization [5], with both its quality and quantity influencing clearance. High LPS-activity is further a risk factor for incident type 2 diabetes and associated with obesity, features of the metabolic syndrome, and the development of kidney disease in patients with type 1 diabetes [6-8].

Patients with type 1 diabetes display an increased gut permeability and decreased gastro-intestinal motility [9,10], and children who develop type 1 diabetes have an altered gut microbiota [11]. Importantly, these factors can directly affect the translocation of LPS through the intestinal epithelium. Furthermore elevated circulating inflammatory cytokines and an increased arterial stiffness is evident in patients with type 1 diabetes in response to acute hyperglycaemia [12]. In addition lipoproteins in the postprandial phase can directly influence the vascular endothelial cells and interfere with their function [13,14]. It should also be noted that most of the previous oral fat-load studies have mainly focused on short term effects of a single fat load [15]. Given this background, we wanted to investigate whether the consumption of three consecutive high-fat meals during one day would affect postprandial endotoxemia, systemic inflammation, the augmentation index, and lipid metabolism in patients with type 1 diabetes and non-diabetic controls. We hypothesized that the three consecutive high-fat meals would lead to an increase in systemic endotoxin levels that would in turn be associated with dyslipidemia, inflammation and vascular dysfunction. Here we report that patients with type 1 diabetes display adverse changes in HDL/chylomicron metabolism and impaired vasodilation in response to three consecutive high-fat meals, which may be early signs of cardiovascular disease. These changes were independent of postprandial endotoxemia.

## Material and methods

The study subjects were recruited by the Finnish Diabetic Nephropathy Study (FinnDiane; http://www.finndiane.fi) and involved 37 patients with type 1 diabetes and 34 non-diabetic controls. All participants were Finnish citizens of Caucasian background. Inclusion criteria for the study were 1) age <65 years; 2) non-smoker; 3) no use of antibiotics during the past month; 4) no trips outside the Nordic countries during the past month. Voluntary controls were recruited from the laboratory personnel. Type 1 diabetes was defined as age at onset below 40 years and permanent insulin treatment initiated within one year of diagnosis. Participants completed a three-day food-record prior to the day of investigation. Dietary intake was analysed as previously described [16]. Information on the use of medication was recorded with a standardized questionnaire. The study protocol was approved by the local ethical committee (Ethics Committee, Department of Medicine, Helsinki University Central Hospital), and all participants gave an informed consent to participate in the study.

Participants were given three energy-rich meals (2600 kcal) during one day: breakfast (at 8:00–965 kcal, 58% of total energy (E%) from fats), lunch (12:00–870 kcal, 44 E% fats), and dinner (16:00–779 kcal, 46 E% fats). Blood samples were drawn after overnight fasting and every two hours until 18:00 hrs. Urine was collected for 24-hours during the research day. Concentrations of blood glucose, urinary albumin, and serum creatinine as well as white blood cell counts (at 8:00 and 18:00) were determined by routine methods at the laboratory of Helsinki University Central Hospital (HUSLAB, Helsinki, Finland). Kidney status and function were assessed by calculating the urinary albumin excretion rate (AER) and the estimated glomerular filtration rate (eGFR). Patients with an AER < 300 mg/24 h were included in the analysis. Serum insulin concentrations were determined with Wallac AutoDELFIA Insulin kit (PerkinElmer, Turku, Finland) using an automatic analyser (Wallac 1235 Automatic Immunoassay System, Wallac, Turku, Finland).

Serum triglycerides, total cholesterol, HDL-cholesterol, apolipoprotein (apo) A-I (kit 981702; Konelab Thermo Fischer, Vantaa, Finland), and apoB-100 (kit 981703; Konelab) concentrations were determined with a Konelab analyser using automated enzymatic methods (Thermo Scientific, Vantaa, Finland). Plasma apoB-48 concentrations were measured using an ELISA kit (AKHB48, Shibayagi Co LtD, Shibukawa, Japan). ApoE concentrations [17], as well as phospholipid transfer protein (PLTP) [18], cholesteryl ester transfer protein (CETP) [19,20], and paraoxonase (PON-1) [21] activities were measured as previously reported. Fasting serum lipid metabolites were analysed by nuclear magnetic resonance (NMR) spectroscopy (NMR Metabolomics Laboratory, Kuopio, Finland) as previously described [22]. The size distribution of the HDL-subclasses was very large HDL (average diameter of 14.3 nm), large HDL (12.1 nm), medium HDL (10.9 nm), and small HDL (8.7 nm).

### LPS-activity

Serum LPS-activity was measured kinetically from 1:5 diluted serum samples with the Limulus amoebocyte lysate assay (LAL, Hycult, Uden, the Netherlands). Briefly, the colour formation at 405 nm was recorded every 2 min, for a total of 40 min. Since a postprandial rise in triglyceride concentrations as well as hemolysis may lead to arbitrarily high endotoxin levels in the LAL assay [23], we subtracted the minimum absorbance value from the maximum value, measuring the relative change of absorbance in each sample.

### Inflammatory factors

High sensitivity CRP (hsCRP kit 981798; Konelab) concentrations were measured from serum samples with a Konelab analyzer using automated enzymatic methods (Thermo Scientific, Vantaa, Finland). Serum amyloid A (SAA), IL6 and soluble CD14 (sCD14) ELISA measurements were performed according to the manufacturer’s instructions (Quantikine Human SAA, Quantikine HS Human IL6, and Quantikine Human sCD14 immunoassays; R&D systems, Abingdon, UK). From each separately run plate a set of controls was chosen and re-run together to normalize inter-assay variation.

### Insulin sensitivity and the augmentation index

In patients with type 1 diabetes, time and dosage of insulin injections were recorded during the investigation day. Insulin requirements, measured in units per kilogram body weight, were used as a surrogate marker of insulin sensitivity [24,25]. In the non-diabetic subjects, the homeostasis model assessment was used to calculate insulin resistance (HOMA-IR) index [plasma insulin (mU/mL) × plasma glucose (mmol/L)/22.5]. Glucose variability in patients with type 1 diabetes was calculated as the standard deviation of all six measurements of the blood glucose between 8:00 and 18:00.

Arterial stiffness was indirectly estimated by the augmentation index (AIx) at fasting and at 12.00 and 16.00. Briefly, applanation tonometry (SphygmoCor, Atcor Medical, Sydney, Australia) is a non-invasive method to estimate arterial stiffness by analyzing arterial pressure waveforms as described earlier [26,27]. The pulse wave was recorded from the radial artery of the right arm with a high-fidelity micromanometer. A model of the central pressure waveform was synthesized by the SphygmoCor software using a validated generalized mathematic transformation to calculate the augmentation index and aortic pulse pressure as indicators of arterial stiffness [28]. AIx was corrected for heart rate.

### Statistics

Between groups with normally distributed parameters variation was analysed with Student’s *t*-test or ANOVA. Non-normally distributed variables were tested with Mann–Whitney U or Kruskal-Wallis tests. To compare distributions at two different time points of non-normally distributed variables Wilcoxon signed rank test was used. Differences in distributions of several time points were analysed with Friedman’s test for several related samples. Pearson’s or Spearman’s correlation analyses were used as appropriate. Using partial correlation, the effect of confounding factors was taken into account.

The postprandial response was defined by the area under the curve (AUC), which was calculated using all time points between 8:00 and 18:00 with the following formula [2 hours * ((x_1_/2) + x_2_ + x_3_ + x_4_ + x_5_ + (x_6_/2)), where x = value at time point]. For the incremental area (IncA) the area below the first time point * 10 hours was subtracted from AUC, unless some other time interval is indicated. All statistical analyses were carried out using SPSS 15.0 (Chicago, Illinois, USA).

## Results

Patients with type 1 diabetes and non-diabetic controls displayed similar age, gender distribution, BMI, blood pressure and blood lipid profiles at baseline (Table 1). Blood pressure lowering medication was used by 38% of the patients and 9% of the controls (p = 0.005), corresponding values for lipid lowering medication were 24% and 3% (p = 0.014). Based on 3-day food records, the mean energy intake did not differ between patients with type 1 diabetes and controls (2090 ± 420 vs. 2097 ± 702 kcal/24 h; Additional file 1: Table S1). Energy derived from dietary fats was also similar between the groups (T1D 41% vs. controls 38%). The energy intake during the 10-hour study was 2600 kcal, of which 50% came from fats. Individual meal composition is shown in Additional file 1: Table S2.

**Table 1:** Clinical characteristics of study participants at fasting

	**Controls**	**Type 1 diabetes**	**p**
**Age (yrs)**	**38.2 ± 10.3**	**42.5 ± 9.4**	**ns**
**Sex (M/F)**	**18/16**	**16/21**	**ns**
**Duration of diabetes (yrs)**	**-**	**26 ± 13**	**-**
**BMI (kg/m**^**2**^**)**	**25.2 ± 4.2**	**26.1 ± 3.6**	**ns**
**Systolic blood pressure (mmHg)**	**129 ± 14**	**133 ± 17**	**ns**
**Diastolic blood pressure (mmHg)**	**77 ± 10**	**77 ± 8**	**ns**
**HbA**_**1c **_**(%) (mmol/mol)**	**5.3 ± 0.3 (34 ± 3.3)**	**8.0 ± 1.3 (64 ± 14.2)**	**<0.001**
**eGFR (ml/min/1.72 m**^**2**^**)**	**102 (93–110)**	**101 (94–114)**	**ns**
**AER (mg/24 h)**	**3.4 (2.4–5.2)**	**7.0 (3.8–13.4)**	**0.001**
**Blood glucose (mmol/l)**	**4.9 ± 0.6**	**8.7 ± 3.4**	**<0.001**
**Cholesterol (mmol/l)**	**4.7 ± 0.7**	**4.6 ± 0.7**	**ns**
**Triglycerides (mmol/l)**	**1.0 ± 0.3**	**0.9 ± 0.5**	**ns**
**HDL-chol (mmol/l)**	**1.3 (1.1–1.7)**	**1.6 (1.3–1.8)**	**ns**
**LDL-chol (mmol/l)**	**2.8 ± 0.7**	**2.6 ± 0.7**	**ns**
**ApoB-100 (mg/dL)**	**83 (73–93)**	**72 (66–87)**	**ns**
**ApoA1 (mg/dL)**	**138 (123–158)**	**153 (132–162)**	**ns**
**ApoB-48 (mg/dL)**	**3.8 (2.7–5.4)**	**5.0 (3.7–8.8)**	**0.014**
**High-sensitive CRP (mg/l)**	**1.0 (0.2–2.7)**	**1.5 (0.3–5.4)**	**ns**
**Interleukine-6 (pg/ml)**	**1.2 (0.8–2.0)**	**1.5 (0.5–3.5)**	**ns**
**LPS (EU/ml)**	**0.82 (0.57–1.43)**	**0.90 (0.52–1.24)**	**ns**

Data at fasting are presented as mean ± SD or median (25^th^-75^th^ quartile). AER – albumin excretion rate, eGFR – estimated glomerular filtration rate.

### Effects of high-fat meals on serum LPS activity levels

**Figure 1:**
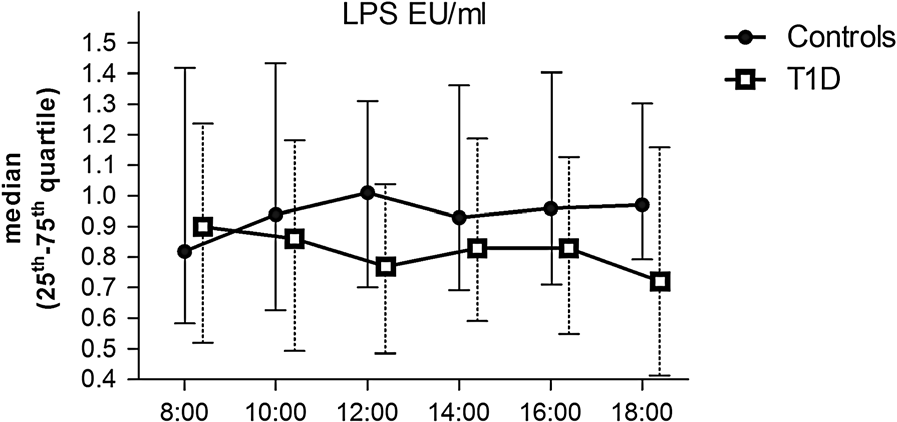
**The effects of three high-fat meals on serum LPS-activity levels.** Non-diabetic controls and patients with type 1 diabetes are indicated with filled circles and open squares, respectively. Lines indicate median and whiskers 25^th^ – 75^th^ quartile. Meals were given at 8:00, 12:00, and 16:00 (time on x-axis).

Fasting LPS and LPS-AUC were comparable in patients with type 1 diabetes and controls [median (25^th^-75^th^ quartile): fasting 0.82 (0.57-1.43) vs. 0.90 (0.52-1.24) EU/ml and AUC 9.8 (6.8-14.1) vs. 8.8 (6.2-11.3)]. Consecutive high-fat meals had only a modest effect on LPS activity (Figure 1) and an increase in activity was seen in a similar proportion of controls and patients. After breakfast (8:00–12:00) the incremental LPS-activity area (IncA) was higher in the controls compared to the patients with type 1 diabetes [0.19 (−0.39-1.00) vs. -0.30 (−0.81-0.32); p = 0.032].

Serum lipids showed a positive association with systemic LPS-activity levels. In controls fasting LPS correlated with triglycerides (r = 0.500; p = 0.004) and ApoB-100 (r = 0.398; p = 0.027). LPS-AUC correlated with cholesterol-AUC (p = 0.386; p = 0.047), LDL-cholesterol-AUC (r = 0.361; p = 0.046), and ApoB-100-AUC (r = 0.489; p = 0.005). In patients with type 1 diabetes both fasting LPS and LPS-AUC correlated with CRP (fasting r = 0.428; p = 0.009, AUC r = 0.338; p = 0.044) and insulin dose (fasting r = 0.479; p = 0.004, AUC r = 0.528; p = 0.001) (Additional file 1: Figure S1). To further characterise this association, we divided patients with type 1 diabetes into tertiles based on their daily insulin dose. Those in the highest tertile had significantly higher LPS-AUC (p for trend 0.003), BMI (p for trend 0.011), systolic and diastolic blood pressure (p for trend 0.030 and 0.005), triglyceride-AUC (p for trend 0.004), apoB-100-AUC (p for tend 0.011), and lower HDL-cholesterol-AUC (p for trend 0.018).

### Food records

To investigate whether pre-study dietary intake is associated with a higher postprandial endotoxin load, food records were analysed for the association between LPS-AUC and energy-, fat-, protein-, and carbohydrate intake adjusted for BMI. However, no associations between LPS-AUC and the dietary intake were found. Neither did LPS-AUC correlate with the intake of mono-, poly- and saturated fatty acids. Fasting LPS, on the other hand, correlated negatively with the BMI-adjusted fat intake in controls (r = −0.375; p = 0.038). The same trend was observed in patients with type 1 diabetes (r = −0.306; p = 0.070).

### Inflammatory markers

IL-6 levels increased throughout the day peaking at 16:00 in both the patients with type 1 diabetes (median increase from fasting: 610% increase) and the controls (570% increase). However, no association between LPS-activity and IL-6 levels was observed. The inflammatory markers CRP, sCD14 and SAA varied only modestly during the day and the patients with type 1 diabetes were no different from the controls. Fasting leukocyte (5.6 vs. 5.4 E9/l) and neutrophil (3.1 vs. 2.8 E9/l) counts were slightly higher in patients with type 1 diabetes compared to controls. Both leukocyte and neutrophil counts increased significantly during the 10-hour long examination day. In patients with type 1 diabetes the increase was blunted: leukocyte delta change (T1D vs. control 114 vs. 128%; p = 0.022), and neutrophil delta change (118 vs. 147%; p = 0.038) between 8:00 and 18:00.

### Augmentation index

**Figure 2:**
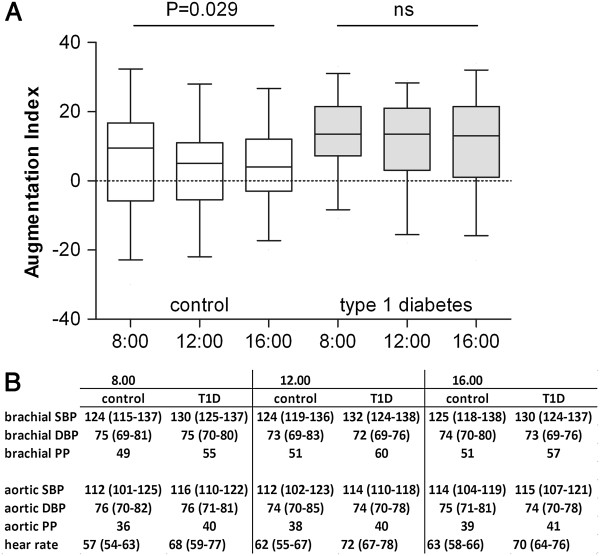
**The effects of three consecutive high-fat meals on arterial stiffness. (A)** The augmentation index was higher postprandially in patients with type 1 diabetes compared to controls (grey bars 12:00; p = 0.020 and 16:00; p = 0.028). In controls the arteries significantly relaxed during the day (p for trend 0.029). **(B)** Bracial and aortic systolic blood pressure (SBP), diastolic blood pressure (DBP), pulse pressure (PP) mmHg and heart rate (beats/min) during the research day.

Patients with type 1 diabetes and controls showed similar augmentation index (AIx) at fasting but at 12:00 and 16:00 the AIx was higher in patients with type 1 diabetes (Figure 2). The augmentation index dropped postprandially in the controls (p for trend 0.029), but the patients with type 1 diabetes showed no such response (Figure 2). However, in patients with type 1 diabetes the glucose-variability during the examination day correlated with the AIx: 8:00 (r = 0.420, p = 0.017), 12:00 (r = 0.392, p = 0.048), and 16:00 (r = 0.434, p = 0.013). No association with LPS-activity was observed.

### Lipid metabolism

Fasting and postprandial triglyceride concentrations were similar between the two groups. Serum triglyceride concentrations increased after the meals peaking at 14:00 and 18:00 (Additional file 1: Figure S2). Fasting triglycerides predicted postprandial triglyceride response in both groups (Additional file 1: Figure S2). Those in the highest fasting triglyceride-tertile had the highest apoB-48-AUC (T1D p for trend <0.001; controls p = 0.003) and chylomicron triglyceride-AUC (p = 0.001; p = 0.004), as well as the lowest HDL-AUC (p = 0.005; p = 0.002) (Additional file 1: Figure S2).

### Chylomicron markers and LPS uptake

**Figure 3:**
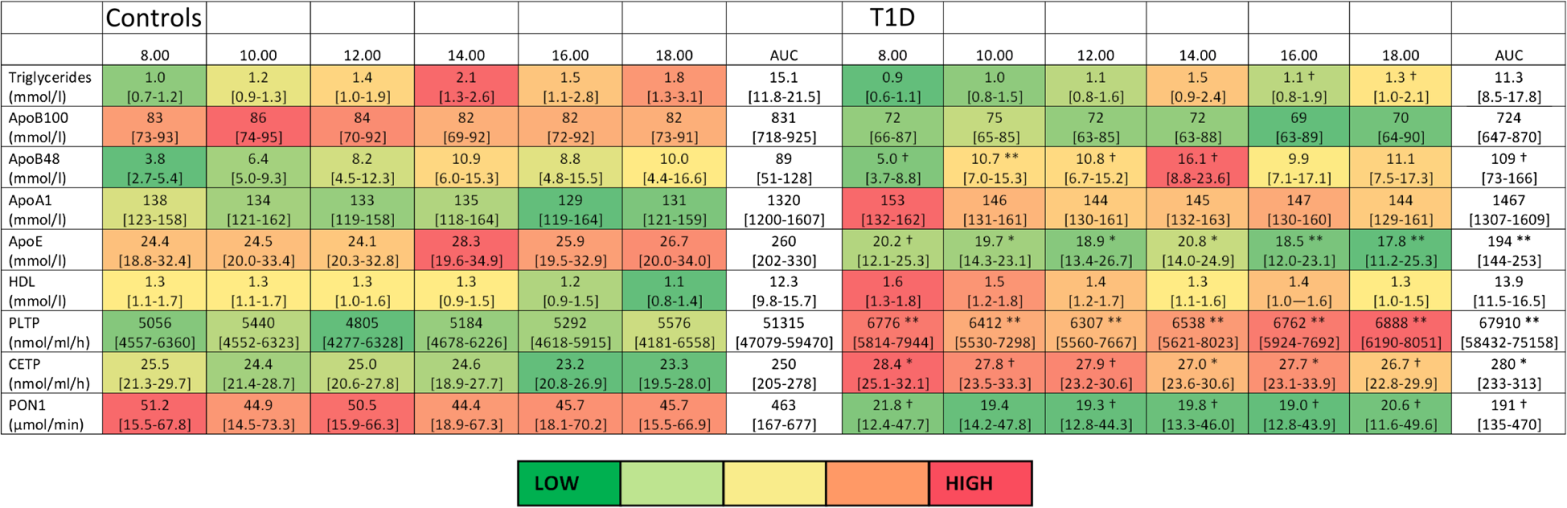
**Impact of three consecutive high-fat meals on serum lipid parameters in non-diabetic controls and patients with type 1 diabetes (T1D).** PLTP - phospholipid transfer protein; CETP - cholesteryl ester transfer protein; PON – Paraoxonase; AUC – area under curve. Data is presented as median [25^th^-75^th^ quartile]. Significance of difference at each time point and in AUC between groups are marked as: **p ≤ 0.001; *p ≤ 0.01; †p ≤ 0.05. Low values are coloured green, high values red.

Our next aim was to evaluate if there was a relationship between chylomicron metabolism and LPS-activity, since chylomicrons have been described to transport lipid-soluble endotoxins from the gut [2,5]. ApoB-48, a specific chylomicron marker, was higher both at fasting [5.0 (3.7-8.8) vs. 3.8 (2.7-5.4 mg/dL); p = 0.014] and after the meals [AUC 109 (73–166) vs. 89 (51–128); p = 0.035] in the patients with type 1 diabetes compared to the controls (Figure 3). In patients with type 1 diabetes apoB-48 correlated positively with the LPS-activity only after the breakfast (8.00-12.00) (apoB-48-IncA vs. LPS-IncA r = 0.351; p = 0.036). Although at fasting the triglyceride-content of the chylomicrons was similar between the groups, patients with type 1 diabetes had lower chylomicron triglyceride concentrations [AUC 1.15 (0.87-1.86) vs. 1.72 (1.10-1.869); p = 0.037] postprandially.ApoE, a regulator of chylomicron turn-over, was lower in patients with type 1 diabetes at fasting [T1D vs. control 20.2 (12.1-25.3) vs. 24.4 (18.8-32.4 mg/dL); p = 0.022] and postprandially [AUC 194 (144–253) vs. 260 (202–330); p = 0.001] (Figure 3). In controls fasting apoE correlated with ApoB-48 (r = 0.463; p = 0.015) and apoE-AUC with apoB-48-AUC (r = 0.451; p = 0.018). These correlations were not seen in patients with type 1 diabetes.

### HDL & LPS detoxification

NMR-analysis was used to determine HDL-size distributions at fasting. Despite similar HDL cholesterol concentrations patients with type 1 diabetes had more large HDL particles (14.3 nm) [441*10^−7^ ± 176*10^−7^ vs. 309*10^−7^ ± 216*10^−7^ nmol/l; p = 0.007] than controls. In patients with type 1 diabetes, the number of small-size (8.7 nm) HDL particles (r = 0.478, p = 0.002) and their total lipid concentration (r = 0.481, p = 0.002) correlated positively with the LPS-activity.Serum lipid transfer proteins involved in HDL-modelling were elevated in patients with type 1 diabetes compared to controls: PLTP-AUC [67910 (58431–75158) vs. 51315 (47079–59470); p < 0.001] and CETP-AUC [280 (233–313) vs. 250 (204–278); p = 0.007] (Figure 3). On the other hand, paraoxonase (PON-1)-AUC, an anti-oxidative enzyme bound to HDL, was lower in patients with type 1 diabetes [191 (135–470) vs. 463 (167–678); p = 0.027] (Figure 3).

## Discussion

We investigated the postprandial effect of three consecutive high-fat meals on endotoxemia, inflammation, the augmentation index, and lipid metabolism in non-diabetic controls and patients with type 1 diabetes. The strengths of our study were that normal eating behaviour was mimicked by multiple meals during the day, a relatively large number of subjects was included, and a vast array of metabolic and physiological variables were investigated in order to characterise the systemic changes. Importantly, and to the best of our knowledge, postprandial endotoxemia was investigated here for the first time in patients with type 1 diabetes. We found that the LPS-activity was only modestly affected by the meals. Patients with type 1 diabetes showed no significant decrease in the augmentation index in response to the high-fat meals and displayed altered chylomicron and HDL metabolism.

Surprisingly, in non-diabetic controls or patients with type 1 diabetes, the three consecutive high-fat meals increased LPS-activity only modestly. Controls displayed elevated LPS-activity after breakfast and higher postprandial triglyceride content in the chylomicrons. The combination of lower LPS-activity and lower chylomicron triglycerides in the patients might be explained by an increased lipid clearance by a high lipoprotein lipase-activity [29] or a decreased lipid absorption from the gut.

A mixed meal containing lipids was previously shown to be associated with an acute increase in endotoxemia and inflammation [2,23]. This contrast to our data may be explained by differences in quantity and quality of fat between study meals. Furthermore, lipemic interference on the LAL assay may introduce a bias not accounted for by previous studies [23]. To overcome this problem we subtracted the sample background from the absorption to correct for lipemia, which may explain why the LPS-activity increased only in a subgroup of the studied individuals during the day. In addition to LPS, free fatty acids also act as ligands for TLR4 and can postprandially trigger inflammation and increase circulating cytokines [30]. No association was observed between serum LPS-activity and inflammatory markers (IL6, SAA, CD14, and CRP). Based on previous studies, intravenously injected LPS seems to have a higher immunostimulatory potency compared to intestinally derived endotoxins after a high-fat diet. This discrepancy could possibly be explained by the efficient dephosphorylation of intestinal LPS-molecules by intestinal alkaline phosphatase [30].

Increased postprandial endotoxemia is associated with markers of insulin resistance [31], probably explaining the strong association between the LPS-AUC and the body weight adjusted daily insulin dose. In fact, LPS-AUC was the highest in those with high CRP and a high insulin dose. A high insulin dose was furthermore associated with elevated BMI, systolic blood pressure, and altered lipid metabolism, supporting an insulin resistant phenotype.

Patients with type 1 diabetes presented higher AIx, a surrogate estimate of arterial stiffness, and were unable to induce relaxation of the arteries in response to the high-fat diet. The strong correlation with glucose variability may partly explain this finding. Inflammation and insulin resistance also cause arterial stiffness although the process is poorly understood. In patients with type 1 diabetes insulin resistance is associated with the inability of insulin to decrease central aortic pressure, possibly predisposing to premature stiffening of large arteries [32]. Similarly, weight loss in patients with type 2 diabetes was shown to decrease arterial stiffness. This was mainly explained by improved insulin sensitivity and a reduction in inflammatory factors [33]. We have shown an acute increase in arterial stiffness and inflammatory markers in patients with type 1 diabetes during a hyperglycaemic clamp [12]. However, in the present study no such correlation was observed between AIx and inflammatory markers nor insulin resistance. Also a direct effect of TRLs on endothelial function is a possible mechanism [13,14], especially as chylomicron remnants were elevated in patients with type 1 diabetes. Nevertheless, a number of other mechanisms such as a deficiency of endothelial vasodilators could have an impact on the endothelial function and the arteries in our study.

The serum triglycerides increased throughout the day in response to the high-fat meals. Interestingly, patients with type 1 diabetes presented conspicuous changes in chylomicron levels. ApoB-48, a chylomicron structural protein and as such a marker of intestinal TRL and their remnants, was elevated in the patients at fasting and throughout the day compared to controls. Interestingly uptake of ApoB (−100 and −48) has been described in leukocytes, leading to activation in response to TRLs [34]. Furthermore an elevation in apoB-48 is considered a risk factor for cardiovascular disease [35,36], and high levels could be caused by a decreased removal of remnants or an increased production of chylomicrons. Another lipoprotein involved in the catabolism of triglyceride rich particles is apoE [37], which is the master regulator of chylomicron remnant turnover and facilitates their binding to their specific hepatic receptor, low density lipoprotein-receptor related protein 1, LRP1 [37]. In support of a defect in the clearance capacity of chylomicron remnants, apoE levels where lower in patients with type 1 diabetes. Moreover, HDL-bound apoE has *in vitro* been shown to induce the activity and stability of the antioxidative enzyme PON-1 [38]. High apoB-48, low apoE, and a decrease in PON-1 activity (anti-oxidative capacity of HDL) together with impaired AIx response suggest that patients with type 1 diabetes are likely at higher risk of vascular dysfunction and cardiovascular disease.

Moreover, HDL has been considered the main detoxifier of circulating endotoxins. The higher proportion of large HDL particles and higher PLTP activity suggests a potentially accelerated LPS clearance in patients [39]. Notably in patients, the small-size HDL particle concentration was positively correlated with LPS-activity. These data suggest differences in the distribution of HDL-particles and their composition between patients with type 1 diabetes and controls. Whether intestinal alkaline phosphatase activity, in addition to potential differences in HDL protective functions, can explain the lower LPS-activity in patients with type 1 diabetes is an open question.

In conclusion, we examined the acute effects of multiple high-fat meals on endotoxemia, inflammation, arterial stiffness, and lipid metabolism in patients with type 1 diabetes and non-diabetic controls. In the patients the high-fat meals were associated with blunted decrease in the augmentation index as well as an elevation of chylomicron remnants (high apoB-48 and low apoE), which may contribute to a higher risk of cardiovascular disease. This effect on the vasculature could not be explained by an increased inflammatory response to the high-fat meals because the increase in IL-6 and circulating leukocytes was similar in the controls and patients. However, differences in factors related to HDL/apoB-containing lipoprotein metabolism (PLTP, CETP and PON-1) suggest that there are changes in HDL-mediated functions such as inflammation, oxidation and reverse cholesterol transport between the two groups. Of note, three consecutive high-fat meals during one day had no significant effect on postprandial LPS-activity levels in non-diabetic subjects or patients with type 1 diabetes. Thus, metabolic endotoxemia may be more central in patients with chronic metabolic disturbances such as obesity, type 2 diabetes, or diabetic kidney disease.

## Competing interest

AJK, PS and MAK are shareholders of Brainshake Ltd, a startup company offering NMR-based metabolite profiling. No potential conflicts of interest relevant to this paper were reported.

## Authors’ contributions

MIL and ML were responsible for data assembly, statistical analysis, and writing the manuscript. DG, V-PM, AJA and SH contributed to data analysis and manuscript revision. ML, MJ, MRT and PJP participated in designing the study and the interpretation of results. CF, JK, OV and JKN contributed to the study design, participated in the interpretation of the results, and critically reviewed the article. CLF and LP collected samples, analysed data and critically reviewed the manuscript. AJK, PS, and MAK conducted the NMR analysis. PHG contributed to the study design and critically reviewed the manuscript; he is also the guarantor for the study. All authors read and approved the final manuscript.

## Supplementary Material

Additional file 1: Figure S1 Association of serum LPS-activity levels with insulin resistance and inflammation in patients with type 1 diabetes. Mean of LPS-AUC in patients with type 1 diabetes divided by median of insulin dose (low ≤0.63; high>0.63 units/kg) and hsCRP (low≤1.5; high>1.5 mg/l). LPS-AUC was increased in patients with a high insulin dose and high hsCRP (low-low vs. high-high p=0.009; p for trend between all groups 0.045). **Figure S2.** Effects of three consecutive high-fat meals on serum triglyceride, HDL, and chylomicron metabolism. Lipid parameters divided by fasting triglyceride tertiles during the study day (time on x-axis) in controls and patients with type 1 diabetes. Mean±SEM. TG-triglycerides; chylo TG –triglyceride content in chylomicrons. ApoB-48-AUC is higher (p=0.035) and chylo TG-AUC lower (p=0.037) in patients with type 1 diabetes compared to controls. **Table S1.** Mean 24h energy and macronutrient intake based on three-day food records. **Table S2.** Meal composition of breakfast, lunch and dinner during the study day. Click here for file
