# Supplementary material for: Patients with type 1 diabetes show signs of vascular dysfunction in response to multiple high-fat meals
Source: Nutr Metab (Lond). 2014 Jun 13;11:28. doi: 10.1186/1743-7075-11-28 (PMC4067102; doi:10.1186/1743-7075-11-28)
Supplement: Additional file 1: Figure S1 — Association of serum LPS-activity levels with insulin resistance and inflammation in patients with type 1 diabetes. Mean of LPS-AUC in patients with type 1 diabetes divided by median of insulin dose (low ≤0.63; high>0.63 units/kg) and hsCRP (low≤1.5; high>1.5 mg/l). LPS-AUC was increased in patients with a high insulin dose and high hsCRP (low-low vs. high-high p=0.009; p for trend between all groups 0.045). Figure S2. Effects of three consecutive high-fat meals on serum triglyceride, HDL, and chylomicron metabolism. Lipid parameters divided by fasting triglyceride tertiles during the study day (time on x-axis) in controls and patients with type 1 diabetes. Mean±SEM. TG-triglycerides; chylo TG –triglyceride content in chylomicrons. ApoB-48-AUC is higher (p=0.035) and chylo TG-AUC lower (p=0.037) in patients with type 1 diabetes compared to controls. Table S1. Mean 24h energy and macronutrient intake based on three-day food records. Table S2. Meal composition of breakfast, lunch and dinner during the study day. [file 1743-7075-11-28-S1.docx]

**Supplemental figure 1.** Association of serum LPS-activity levels with insulin resistance and inflammation in patients with type 1 diabetes. Mean of LPS-AUC in patients with type 1 diabetes divided by median of insulin dose (low ≤0.63; high>0.63 units/kg) and hsCRP (low≤1.5; high>1.5 mg/l). LPS-AUC was increased in patients with a high insulin dose and high hsCRP (low-low vs. high-high p=0.009; p for trend between all groups 0.045).


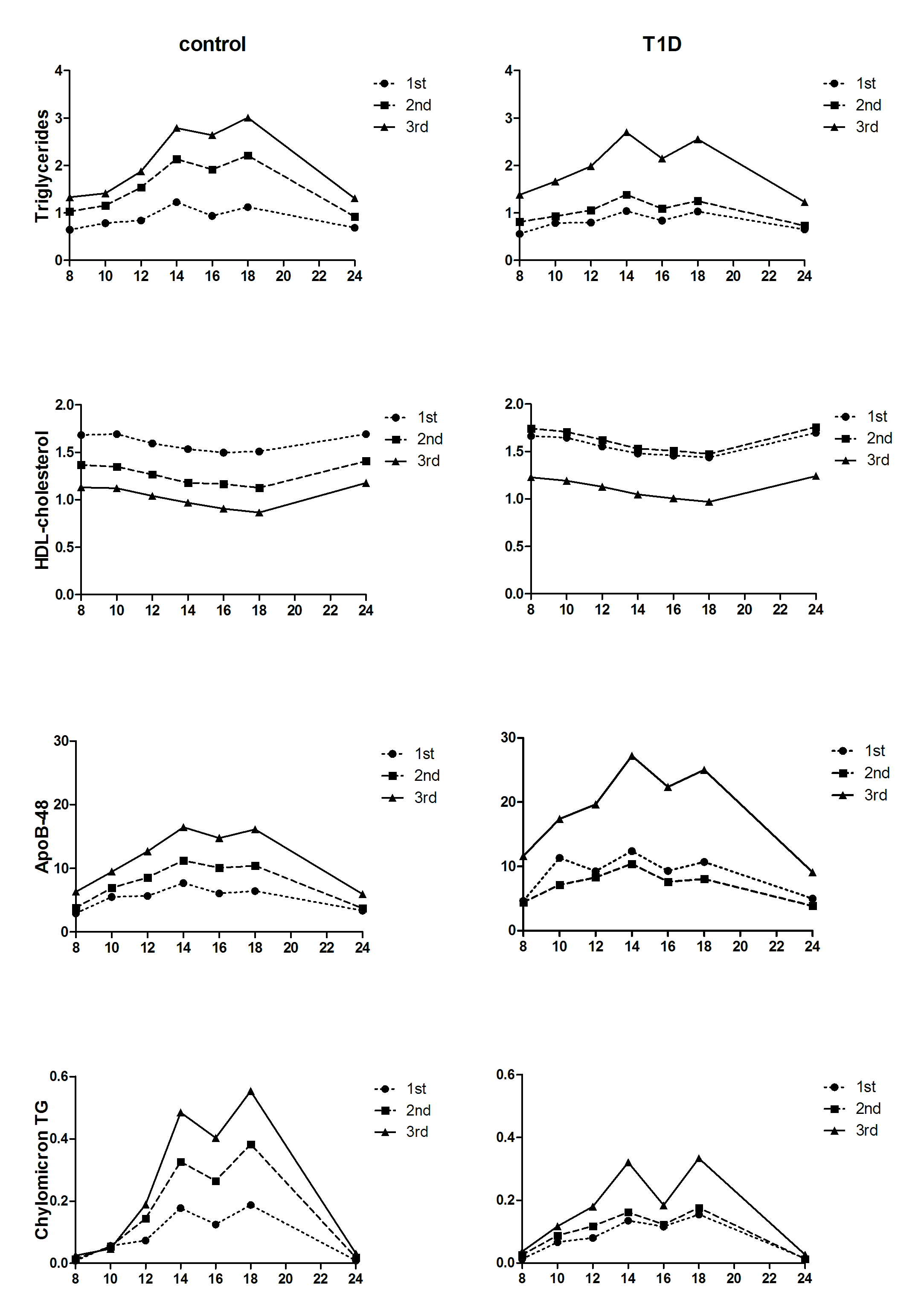


**Supplemental figure 2.** Effects of three consecutive high-fat meals on serum triglyceride, HDL, and chylomicron metabolism. Lipid parameters divided by fasting triglyceride tertiles during the study day (time on x-axis) in controls and patients with type 1 diabetes. Mean±SEM. TG-triglycerides; chylo TG –triglyceride content in chylomicrons. ApoB-48-AUC is higher (p=0.035) and chylo TG-AUC lower (p=0.037) in patients with type 1 diabetes compared to controls.

**Supplemental table 1**. Mean 24h energy and macronutrient intake based on three-day food records.

|  | **Controls** | **T1D** | **All** | **Test meal (10h)** |
| --- | --- | --- | --- | --- |
| Energy (kcal) | 2091 ± 420 | 2098 ± 703 | 2094 ± 583 | 2600 |
| Protein (%) | 17.0 | 17.1 | 17.0 | 15.1 |
| Carbohydrates (%) | 43.1 | 39.4 | 41.2 | 31.1 |
| Fat (%) | 38.0 | 41.3 | 39.8 | 50.1 |
| Monounsaturated fat (%) | 12.8 | 13.8 | 13.3 | 14.8 |
| Polyunsaturated fat (%) | 6.4 | 6.6 | 6.5 | 3.7 |
| Saturated fat (%) | 13.4 | 14.9 | 14.2 | 23.5 |

**Supplemental table 2.** Meal composition of breakfast, lunch and dinner during the study day

| **Meal** | **Composition** | **Fat (g)** | **Monounsaturated fat (E%)** | **Polyunsaturated fat (E%)** | **Saturated fat (E%)** |
| --- | --- | --- | --- | --- | --- |
| Breakfast | Bacon, eggs, carrot roll, butter, cheese, pineapple juice | 62 | 20.2 | 4.3 | 20.9 |
| Lunch | Meatballs, gravy, potatoes, pea-pepper-corn mix, pastasallad, tomato, bread, butter, milk, ice-cream | 42 | 12.6 | 3.8 | 23.5 |
| Dinner | Creamy salmon soup, bread, butter, milk, fruit quark | 40 | 10.6 | 2.9 | 26.6 |

**The Finnish Diabetic Nephropathy Study Centers**

| Anjalankoski Health Center | S.Koivula, T.Uggeldahl |
| --- | --- |
| Central Finland Central Hospital, Jyväskylä | T.Forslund, A.Halonen, A.Koistinen, P.Koskiaho, |
|  | M.Laukkanen, J.Saltevo, M.Tiihonen |
| Central Hospital of Åland Islands, Mariehamn | M.Forsen, H.Granlund, A.-C.Jonsson, B.Nyroos |
| Central Hospital of Kanta-Häme, Hämeenlinna | P.Kinnunen, A.Orvola, T.Salonen, A.Vähänen |
| Central Hospital of Kymenlaakso, Kotka | R.Paldanius, M.Riihelä, L.Ryysy |
| Central Hospital of Länsi-Pohja, Kemi | H.Laukkanen, P.Nyländen, A.Sademies |
| Central Ostrobothnian Hospital District, Kokkola | S.Anderson, B.Asplund, U.Byskata, P.Liedes, |
|  | M.Kuusela, T.Virkkala |
| City of Espoo Health Center: |  |
| Espoonlahti | A.Nikkola, E.Ritola |
| Tapiola | M.Niska, H.Saarinen |
| Samaria | E.Oukko-Ruponen, T.Virtanen |
| Viherlaakso | A.Lyytinen |
| City of Helsinki Health Center: |  |
| Puistola | H.Kari, T.Simonen |
| Suutarila | A.Kaprio, J.Kärkkäinen, B.Rantaeskola |
| Töölö | P.Kääriäinen, J.Haaga, A-L.Pietiläinen |
| City of Hyvinkää Health Center | S.Klemetti, T.Nyandoto, E.Rontu, S.Satuli-Autere |
| City of Vantaa Health Center: |  |
| Korso | R.Toivonen, H.Virtanen |
| Länsimäki | R.Ahonen, M.Ivaska-Suomela, A.Jauhiainen |
| Martinlaakso | M.Laine, T.Pellonpää, R.Puranen |
| Myyrmäki | A.Airas, J.Laakso, K.Rautavaara |
| Rekola | M.Erola, E.Jatkola |
| Tikkurila | R.Lönnblad, A.Malm, J.Mäkelä, E.Rautamo |
| Heinola Health Center | P.Hentunen, J.Lagerstam |
| Helsinki University Central Hospital, Department of |  |
| Medicine, Division of Nephrology | A.Ahola, M.Feodoroff, D.Gordin, O.Heikkilä, K.Hietala, J.Kytö, S.Lindh, |
|  | K.Pettersson-Fernholm, A.Sandelin, L.Thorn, J.Tuomikangas, T.Vesisenaho, J.Wadén |
| Herttoniemi Hospital, Helsinki | V.Sipilä |
| Hospital of Lounais-Häme, Forssa | T.Kalliomäki, J.Koskelainen, R.Nikkanen, |
|  | N.Savolainen, H.Sulonen, E.Valtonen |
| Hyvinkää Hospital | L. Norvio, A. Hämäläinen |
| Iisalmi Hospital | E.Toivanen |
| Jokilaakso Hospital, Jämsä | A.Parta, I.Pirttiniemi |
| Jorvi Hospital, Helsinki University Central Hospital | S.Aranko, S.Ervasti, R.Kauppinen-Mäkelin, |
|  | A.Kuusisto, T.Leppälä, K.Nikkilä, L.Pekkonen |
| Jyväskylä Health Center, Kyllö | K.Nuorva, M.Tiihonen |
| Kainuu Central Hospital, Kajaani | S.Jokelainen, K.Kananen, M.Karjalainen, P.Kemppainen, A-M.Mankinen, A.Reponen |
|  | M.Sankari |
| Kerava Health Center | H.Stuckey, P.Suominen |
| Kirkkonummi Health Center | A.Lappalainen, M.Liimatainen, J.Santaholma |
| Kivelä Hospital, Helsinki | A.Aimolahti, E.Huovinen |
| Koskela Hospital, Helsinki | V.Ilkka, M.Lehtimäki |
| Kotka Health Center | E.Pälikkö-Kontinen, A.Vanhanen |
| Kouvola Health Center | E.Koskinen, T.Siitonen |
| Kuopio University Hospital | E.Huttunen, R.Ikäheimo, P.Karhapää, P.Kekäläinen, |
|  | M.Laakso, T.Lakka, E.Lampainen, L.Moilanen, |
|  | L.Niskanen, U.Tuovinen, I.Vauhkonen, E.Voutilainen |
| Kuusamo Health Center | T.Kääriäinen, E.Isopoussu |
| Kuusankoski Hospital | E.Kilkki, I.Koskinen, L.Riihelä |
| Laakso Hospital, Helsinki | T.Meriläinen, P.Poukka, R.Savolainen, N.Uhlenius |
| Lahti City Hospital | A.Mäkelä, M.Tanner |
| Lapland Central Hospital, Rovaniemi | L.Hyvärinen, K.Lampela, S.Pöykkö, T.Rompasaari, S.Severinkangas, T.Tulokas |
| Lappeenranta Health Center | P. Erola, L. Härkönen, P.Linkola, I.Pulli, E.Repo |
| Lohja Hospital | T.Granlund, K.Hietanen, M.Porrassalmi, M.Saari, T.Salonen, M.Tiikkainen, |
| Länsi-Uusimaa Hospital, Tammisaari | I.-M.Jousmaa, J.Rinne |
| Loimaa Health Center | A.Mäkelä, P.Eloranta |
| Malmi Hospital, Helsinki | H.Lanki, S.Moilanen, M.Tilly-Kiesi |
| Mikkeli Central Hospital | A.Gynther, R.Manninen, P.Nironen, M.Salminen, |
|  | T.Vänttinen |
| Mänttä Regional Hospital | I.Pirttiniemi, A-M.Hänninen |
| North Karelian Hospital, Joensuu | U-M.Henttula, P.Kekäläinen, M.Pietarinen, |
|  | A.Rissanen, M.Voutilainen |
| Nurmijärvi Health Center | A.Burgos, K.Urtamo |
| Oulaskangas Hospital, Oulainen | E.Jokelainen, P-L.Jylkkä, E.Kaarlela, J.Vuolaspuro |
| Oulu Health Center | L.Hiltunen, R.Häkkinen, S.Keinänen-Kiukaanniemi |
| Oulu University Hospital | R.Ikäheimo |
| Päijät-Häme Central Hospital | H.Haapamäki, A.Helanterä, S.Hämäläinen, |
|  | V.Ilvesmäki, H.Miettinen |
| Palokka Health Center | P.Sopanen, L.Welling |
| Pieksämäki Hospital | V.Sevtsenko, M.Tamminen |
| Pietarsaari Hospital | M-L.Holmbäck, B.Isomaa, L.Sarelin |
| Pori City Hospital | P.Ahonen, P.Merisalo, E.Muurinen, K.Sävelä |
| Porvoo Hospital | M.Kallio, B.Rask, S.Rämö |
| Raahe Hospital | A.Holma, M.Honkala, A.Tuomivaara, R.Vainionpää |
| Rauma Hospital | K.Laine, K.Saarinen, T.Salminen |
| Riihimäki Hospital | P.Aalto, E.Immonen, L.Juurinen |
| Salo Hospital | A.Alanko, J.Lapinleimu, P.Rautio, M.Virtanen |
| Satakunta Central Hospital, Pori | M.Asola, M.Juhola, P.Kunelius, M.-L.Lahdenmäki, |
|  | P.Pääkkönen, M.Rautavirta |
| Savonlinna Central Hospital | T.Pulli, P.Sallinen, M.Taskinen, E.Tolvanen, T.Tuominen |
|  | H.Valtonen, A.Vartia, S-L.Viitanen |
| Seinäjoki Central Hospital | E.Korpi-Hyövälti, T.Latvala, E.Leijala |
| South Karelia Central Hospital, Lappeenranta | T.Ensala, E.Hussi, R.Härkönen, U.Nyholm, J.Toivanen |
| Tampere Health Center | A.Vaden, P.Alarotu, E.Kujansuu, H.Kirkkopelto-Jokinen, |
|  | M.Helin, S.Gummerus, L.Calonius, T.Niskanen, T.Kaitala, |
|  | T.Vatanen |
| Tampere University Hospital | I.Ala-Houhala, R.Kannisto, T.Kuningas, P.Lampinen, M.Määttä, |
|  | H.Oksala, T.Oksanen, A.Putila, H.Saha, K.Salonen, H.Tauriainen, |
|  | S.Tulokas |
| Tiirismaa Health Center, Hollola | T.Kivelä, L.Petlin, L.Savolainen |
| Turku Health Center | A.Artukka, I.Hämäläinen, L.Lehtinen, E.Pyysalo, H.Virtamo, M.Viinikkala, M.Vähätalo |
| Turku University Central Hospital | K.Breitholz, R.Eskola, K.Metsärinne, U.Pietilä, |
|  | P.Saarinen, R.Tuominen, S.Äyräpää |
| Vaajakoski Health Center | K.Mäkinen, P.Sopanen |
| Valkeakoski Regional Hospital | S.Ojanen, E.Valtonen, H.Ylönen, M.Rautiainen, |
|  | T.Immonen |
| Vammala Regional Hospital | I.Isomäki, R.Kroneld, L.Mustaniemi, M.Tapiolinna-Mäkelä |
| Vasa Central Hospital | S.Bergkulla, U.Hautamäki, V-A.Myllyniemi, I.Rusk |
